# Nosocomial Transmission of Mycobacterium tuberculosis in an Oncological Setting

**DOI:** 10.1017/ash.2024.257

**Published:** 2024-09-16

**Authors:** Sarah Murray, Mery Mustafa, Shauna Usiak, Mini Kamboj

**Affiliations:** Memorial Sloan Kettering Cancer Center

## Abstract

**Objective:** Mycobacterium tuberculosis (MTB) is a contagious airborne disease that is spread from person to person via particles in the air which are expelled when speaking or coughing1. This retrospective observational study aims to assess the nosocomial transmission of pulmonary MTB among inpatient roommates in a high-risk oncological population over a 14-year period. With limited studies on the transmissibility of MTB in such environments, the investigation focuses on evaluating the risk of nosocomial transmission and implementation of appropriate infection control measures. **Design:** A retrospective analysis from 2010 – April 2023 was conducted in an acute care, 500-bed oncological center. Following exposure workups performed by the Department of Infection Prevention and Control, 17 of 57 identified patients with active pulmonary MTB had inpatient stays with roommates. Source infectivity showed 7 AFB smear positive results, 4 MTB PCR positive results, and 14 MTB culture positive results. Some index patients had a combination of AFB, PCR and/or culture positivity. A high-risk exposure is defined as any patient who shared a room with an index patient for >4 cumulative hours during the infectious period. Infectious period was determined for each index patient based on the onset of symptoms and laboratory results. Workups identified 33 exposed roommates who were notified and advised to undergo testing, employing QuantifERON (QFT-GIT) serum test or Tuberculin skin (TST) PPD test at least 8 weeks following their last day of exposure. The overlap between inpatient roommates and index patients ranged from 1 to 4 days, averaging 1.5 days. **Results:** Of the 33 high-risk roommates, 14 (42%) patients were unable to provide follow-up testing for various reasons including: patient expiration prior to testing, patient transfer to hospice, and being lost to follow up. Nineteen (58%) patients completed post-exposure testing. 12 patients underwent PPD testing (63%) and 7 patients underwent QuantifERON testing (37%). Zero (0%) were found to have a positive QuantifERON or PPD following their exposure. 15.8% (N=3) of exposed patients had hematologic malignancies, and 84.2% (N=16) of exposed patients had solid tumor malignancies. **Conclusion:** The risk of active pulmonary MTB transmission in an oncological, inpatient setting was determined to be low. The absence of positive conversions among roommates of confirmed MTB patients underscores the effectiveness of infection control measures, emphasizing the importance of isolating confirmed or suspected cases promptly. Ongoing efforts should continue to focus on these preventive measures to mitigate the risk of MTB transmission in similar high-risk settings.

**References:** 1. How TB Spreads. CDC, 2023. https://www.cdc.gov/tb/topic/basics/howtbspreads.htm

